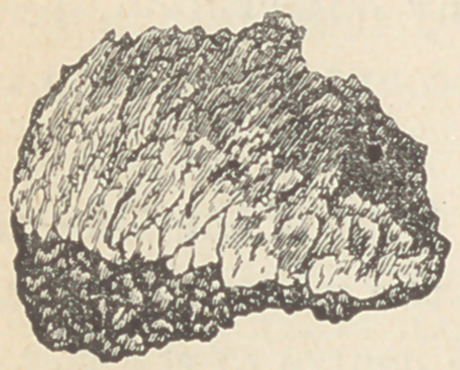# The History of a Case of Gunshot Wound of the Bladder

**Published:** 1878-05

**Authors:** Franklin Staples

**Affiliations:** Winona, Minn.


### THE HISTORY OF A CASE OF GUNSHOT WOUND OF THE BLADDER.

By Franklin Staples, M. D., Winona, Minn.

In part second, Surgical volume, of the Medical and Surgical History of the War of the Rebellion, page 265, under the general head of wounds of the bladder, and among cases reported as having recovered with persistent urinary fistula, is the following report :

" Case 781.—Private R. Butchers, Co. H., 72d New York, aged 20 years, was wounded at Mine Run, Va., Nov. 27, 1863, and was treated on the field until Dec. 5th, and then transferred to hospital at Alexandria. Surgeon E. Bently, U. S. V., reported the case as a gunshot wound of the bladder mainly on the left side; ball removed on the field; simple dressings; discharged the service Oct. 14, 1864; disability total." The history of the case continues and the following appears in the pension examiner's report of examination Sept. 4, 1873, nine years after the soldier's discharge: "There is a large fistulous opening in the urethra in front of the scrotum, also a sinus behind the scrotum on the right side of the perineum which communicates with the bladder. There is evident necrosis of pelvice bones."

History of the case is continued in report of pension examination made Sept. 4, 1875, two years later. The description is as in last report, with addition as follows: "There is a recent fistulous opening in right gluteal region. This has occurred since the last examination."

The Biennial examination of pension examiner Sept. 4, 1877, gives, in addition to what has been given above, the following: "There is now a fistulous opening on the right side of the anus one and a half inches from that described above as on the right of the perineum, back of scrotum. Urine frequenly escapes from both these fistulae."

The object of this communication is to give the continuation of the history of this case as it has come under my observation, and to supply some facts wanting in the above account which help to render the case one of importance.

The ball entered the left groin, passed in front of the femoral vessels and nerve, entered the pelvis through the obturator foramen, passed through the bladder, through the centre of the ischium on the opposite side, lodged in the gluteal muscles on the right side and was removed therefrom by operation on the field. Ten months in the hospital at Alexandria, the first two or three months in bed with a catheter kept constantly in the bladder; urine discharged at each contraction of the bladder through the opening of the exit of the ball, an occasional discharge of small fragments of bone from same opening, with, at length, a cessation of these discharges, and for a time a complete closure of this opening, with patient apparently nearly well, brings the case to the time of his discharge from the service. During the early part of the hospital treatment, however, while wearing the catheter, inflammation and abscess resulted in the large fistulous opening in the urethra in front of the scrotum. From the time of discharge from the service, Oct., 1864, to the autumn of 1871, no discharge from the wound, nor unnatural discharge from the bladder noticed, except at one time in 1868, after lifting he passed a little blood; the bladder sometimes slightly irritable with a tendency at times to void urine rather oftener than usual, the patient in fair health and able to work hard, makes up the history for these seven years. While riding on horseback in the fall of 1871, he first felt a painful swelling in the perineum; abscess, discharge and a permanent fistula resulted. The farther history is given in the reports of the pension examiner above, and extends through a period of seven years, during which time there were two perineal fistulae, besides the urethral fistula in front of the scrotum, with an occasional slight eruption from the old wound of exit in the gluteal region. He has been able to work on a farm during most of this time. On the 11th of Dec., 1877, a detached fragment was removed through the perineum by operation. It was evidently from the inner table of the body of the ischium on the right side, through which bone, the ball passed. Its size and appearance are shown in this cut.

In removing it, the two perineal fistulae were united by incision, making an external opening in size and location not unlike what would be made for the lateral operation for stone, only on the right side. The fistulous tract was followed and the fragment was found to be lodged above the deep perineal fascia near the prostate gland, where it had descended from its place of detachment. Another fragment of bone was now discovered evidently enveloped by a firm fibrinous deposit, and adhesions involving the prostate gland. It had caused a small opening into the prostatic portion of the urethra and sometimes could be felt by the sound as it passed this point. Its removal was considered impossible without inflicting a greater injury than its presence seemed likely to cause, the whole of the prostate gland being involved in what seemed to be a fibrinous deposit around the foreign body. At the present time, two months after the removal of the piece of bone, the opening in the perineum has almost closed, and a little sense of fulness and irritation in the region of the neck of the bladder and prostate gland is now the only source of discomfort.

That such a foreign body as a necrosed spicula of bone could work its way from a distance, and, in its incomplete attempt to make its escape from the body, should, with so little harm, remain lodged in the substance of the prostate body, is worthy of record. Another point in the case is, that for nine years these fragments of bone, although detached at the time of the gun-shot wound, remained in their places without causing any suppurative inflammation, and at length becoming dislodged, abcess and fistulæ resulted. The whole case is one illustrative of nature's ability to guard against damage and repair injury.

---

The Bureau County Circuit court, at Dover, Ill., recently disposed, in the March term, of a suit for damages instituted by a Miss Sansom, of Malden, against Peter Martin, formerly of the firm of Hopkins and Martin, druggists of that place. It was proven in court that the lady sent a small boy to the apothecary's for some arsenic, which she had been in the habit of taking for neuralgia. Soon after swallowing the usual dose of the material sent her by the druggist, the plaintiff became seriously ill, and was near dying in convulsions. She swore that these symptoms have since recurred on several occasions. The medicine was proved to be strychnia instead of arsenic. Suit was instituted, as before stated, and a verdict of damages awarded the plaintiff to the amount of $1,050. Such is the just recompense for the ignorance of a man who, in order to sell liquors, pursued the business of a druggist, and who, when upon the witness stand, could not tell how many ounces made a pound of apothecaries' weight.

## Figures and Tables

**Figure f1:**